# Otherness, Cloning, and Morality in John Wyndham’s *The Midwich Cuckoos* (1957)

**DOI:** 10.1007/s10912-021-09708-z

**Published:** 2021-09-04

**Authors:** Solveig Lena Hansen

**Affiliations:** 1 Faculty 11: Human and Health Sciences, University of Bremen, Bremen, Germany; 2 Department of Medical Ethics and History of Medicine, University Medical Center, Göttingen, Germany

**Keywords:** Otherness, Science fiction, Reproductive technologies, Great Britain, Utilitarianism

## Abstract

The British writer John Wyndham (1903–1969) explored societal effects of surprising or mystical events. A paradigmatic example is *The Midwich Cuckoos* (1957), which portrays identical-looking children born without sexual intercourse. I propose a reading strategy that focuses on the fictional spatial order and analyses how the construction of the children’s otherness interferes with the village’s demarcation. Furthermore, I interpret the mysterious pregnancies as a reference to basic embryo research in the 1950s – cloning. Finally, I scrutinize Wyndham’s negotiation of utilitarianism throughout the novel and his critique of truly utilitarian decisions that are based on constructions of Otherness.

## Introduction

Research on the British writer John Wyndham^1^ (1903–1969) is rare. This comes as a surprise, because some of his post-war works are well known. His novel, *The Chrysalids* (Wyndham [1955] 2008), for instance, was turned into a BBC radio broadcast twice in 1982 and 2012. His particularly famous novel is *The Day of the Triffids* (Wyndham [1951] 1984), which became a classic of British science fiction through its multiple adaptations.^2^ But, besides a small community of researchers, above all Ketterer (2001; 2005) and most recently Määttää (2017; 2020), John Wyndham is considered “the unread bestseller” (Rebellato 2010).

In medical and health humanities, his name may be unknown for two reasons: first, Wyndham’s works were published two decades before the rise of academic and political discourses in applied ethics. In contrast, contemporary authors like Margaret Atwood are interpreted much more often. Second, it was only five years ago that Miller and McFarlane argued that we should consider “the ways in which science fiction proposes concrete alternatives to hegemonic narratives of medical progress and fosters critical self-awareness of the contingent activity which gives ‘the future’ substance in the here-and-now” (2016, 213). This quote illustrates that for a long time in medical and health humanities, science fiction may have been too far-fetched for ethical analysis. Following Miller and McFarlane, I will interpret Wyndham’s novel *The Midwich Cuckoos* ([1957] 1980) against an ethical background, critically negotiating a utilitarian perspective, and against a scientific background, which emerged in the 1950s (i.e. by cloning frogs). Both topoi are, as I will show, connected through Otherness as one of the central elements in science fiction (Roberts 2006, 17; Seed 2011, 27).

Throughout my analysis, I will follow Ketterer’s understanding of Wyndham’s fiction as “logical fantasies” or “reasoned fantasies” (2005, 178). Such “logical fantasies” are characterized by a combination of archetypical features of classical sci-fi, such as the exploration of new technologies and their effects on society, and mystical or fantastic elements:

He wrote SF, a form which aims at the effects of realism while taking as its main plot device – its generic marker – an invention which is *known* not to be real and iconically so signaled. However, no work of SF can be of lasting value and interest unless it is in fact ultimately about something that is real, something of genuine concern to human beings. (Ketterer 2001, 147)

By bringing together advanced technologies and fantasy in an everyday life perspective, Wyndham’s novels seem plausible in the way societies are depicted although some parts of the novel are unrealistic. Through this deviation from reality, Wyndham “offers works rich in symbolic potential” (Link 2015, 63), in which he morally and politically reflects on ideologies of his time: “As he blends Cold-War politics and imaginative elements, Wyndham consistently reaches to images from natural selection, in a bid to awaken in the reader the principles of survival, which have been momentarily usurped by a massified society that rewards weakness” (63) In what follows, the representation of technology is constitutive of many Wyndham plots, but it does not make up its central theme. The focus thus is not on technological progress itself but on the way characters and society are affected by it. This is a characterization also applies to the piece that Ketterer (2011, 374) calls “his best novel, *The Midwich Cuckoos*.^3^.

## Building a fictional microcosm: the spatial order of Midwich

The novel’s narrator, Richard Gayford and his wife, Janet, live a placid life in Midwich. Their life is, in fact, so quiet that Gayford tells the reader he would not be surprised if there “had […] been posts at the entrances to the village bearing a red triangle and a notice below: MIDWICH DO NOT DISTURB” (MC 11). Representing the prototypical utopian island, Midwich is a microcosm of its own, sealed off the rest of the world, as the heading of the chapter frames the novel: “*No Entry to Midwich”* (MC 9). From the beginning, the village is introduced as a peaceful, quiet place with little contact to an unknown, mostly irrelevant, outside. The relevance and the symbolic meaning of its spatial structure becomes even more evident when the village’s topography is introduced:

At the heart of Midwich is a triangular Green ornamented by five elms and a white-railed pond. The war memorial stands in the churchward corner of the Green, and spaced out round the sides are the church itself, the vicarage, the inn, the smithy, the post office, Mrs Welt’s shop, and a number of cottages. Altogether, the village comprises some sixty cottages and small houses, a village hall, Kyle Manor, and The Grange. (11)

While most of these buildings can be expected in a village or small town, three of them stand out. The green triangle can be read as a reference to female reproductivity, representing the color of fertility as well as the vulva. The reference of the war memorial indicates that the story unfolds in a period after World War II. The function of The Grange (originally a regular farm) is most unexpected since “the Ministry took it over for Research” (12). Since The Grange’s interior is inaccessible to all non-scientists, such as the narrator, we never gain insight into the laboratories or experiments. This depiction underscores Wyndham’s strategy of representing science and technology as the backdrop for the plot but not as its central theme: The Grange is introduced parenthetically although, from a certain point of view, it is highly involved in the events.

Though Midwich’s spatial order becomes evident very early in the novel, it becomes even more relevant after what is called the ‘Dayout’ (61). When Richard and Janet return from a journey, they find the village inaccessible, resting under an invisible cupola with a radius of two miles. Everyone in the zone is unconscious. Midwich is then considered an “affected area” (37) although no one knows why or by whom it has been affected. The carefully uttered comment, “There’s The Grange,” (38) by an unknown protagonist, neither provokes any reaction nor provides an explanation.

The novel features a fantasy element here when the “prosaic English village” (175) finds itself in a strange situation reminiscent of the sleeping phase in the Grimm Brothers’ *Sleeping Beauty.* The results are more drastic, however, because several weeks later, “inexplicable pregnancies” (61) occur in the village. They affect all women of childbearing age independent of sexual intercourse. The children are born with supernatural abilities, such as the power to determine the inhabitants’ actions to share knowledge telepathically, thereby also forming a collective identity (122).

Although the concept of gestational surrogacy was possible only in the mid 1980s, decades after *The Midwich Cuckoos* was published (Patel et al. 2018), Wyndham’s scenario can be considered a form of traditional surrogacy. However, the ‘father(s)’ of the Midwich’s cuckoos remain undetected until the story’s end. After all, these surrogate mothers were impregnated unwillingly in what amounts to a type of rape (Ketterer 2011), since their bodies have become “victims of an imposition” (MC 106). The children are described as ‘cuckoos,’ which is an allegory for a species bringing another ‘strange’ species to life. Unlike surrogate mothers, however, the Midwich women are required to keep their babies constituting another imposition. Interestingly enough, abortion is not an option for the women, perhaps the result of it being both a taboo and a criminal act during the time the novel published.^4^

The pregnancies become a serious social problem particularly for unmarried women (MC 62f.). Later in the plot, despite keeping the pregnancies hidden from the outside, the villagers themselves begin to accept the unmarried pregnant women. However, as a form of taking care of these women, keeping the press at bay is most important for Midwich, in order to decrease anxiety and keep the secret of the village. Consequently, the mothers must remain discreet about their situation: “‘You must all know how the cheap papers seize upon anything to do with birth, particularly anything unusual. They make a peepshow of it, as if the people concerned were freaks in a fairground. The parents’ lives, their homes, their children, are no longer their own’” (71). Thus, this special form of reproduction has to be kept secret: ‘[W]e must, every one of us, resolve not to mention, or even hint outside the village, at the present state of affairs’ (72). This special situation, as unexpected as it is, creates a space of social affiliation and acceptance that is separated from all surrounding communities: “It means that a newspaper is unlikely to get anything to go on unless it is directly informed by someone inside the village” (82). Everybody beyond Midwich’s borders who might receive information about the pregnancies is considered a threat to the community.

Being part of the village as well as an unaffected witness of the Dayout, Richard observes Midwich and reports all remarkable events to the local military. Bernard Wescott, the army personnel who mediates between the military and the village, asks Richard to observe the village because of his insider position: “I want a regular report on Midwich’s state of health, mind, and morale so that I can keep a fatherly eye on it. […] I want it so that I can act for Midwich’s benefit, should it be necessary” (51). Richard’s observations make up the main part of the story, which he calls “Midwich’s story” (53) as a means of telling the villagers’ experiences in the localized society. Richard narrates from a perspective marked by a relatively high degree of distance to the events, which is a rare effect given that it is told in the autodiegetic mode (i.e. the first-person perspective). This narrative distance has the interesting effect of a quasi-personification of the village itself – Midwich almost becomes an individual character with emotions and a health status as represented by Richard. Through his monitoring, the village seems to be a self-sufficient subject with the ‘Children’ as its dark side.

## The children as ‘other’

Broadly speaking, the topic of otherness can be scrutinized from two perspectives, which are relevant for the interpretation of *The Midwich Cuckoos*. The first notion of otherness is a psychoanalytical one, interpreting “strangers, gods and monsters […] as tokens of fracture within the human psyche” (Kearney 2003, 4). As Kearney notes, such literary figures of the other “speak to us of how we are split between conscious and unconscious, familiar and unfamiliar” (4). Following a psychoanalytical interpretation of *The Midwich Cuckoos*, Bruhm argues that these “children are not ‘children’ but the representation of the primary narcissistic fantasy of Children” (2016, 170). They remind us of the pleasure of raising expectations towards the other (and children in particular) and transferring expectations and desire towards them without recognizing their own identity.

The second notion of otherness is a political one, which aligns with postcolonial studies. Postcolonial scholars argue that “[t]he existence of others is crucial in defining what is ‘normal’ and in locating one’s own place in the world” (Ashcroft, Griffiths, and Tiffin 2007, 154). Here, ‘Other’ “can refer to the colonized others who are marginalized by imperial discourse, identified by their difference from the centre and, perhaps crucially, become the focus of anticipated mastery by the imperial ‘ego’” (155).

For both notions of otherness, the category of space is relevant. In the psychoanalytic and phenomenological tradition, space is the central category to differentiate subjects being-in-the world from other subjects. It allows one to discern one’s identity as separate from one’s own (Pinsky 2003). Thus, space prerequisites responsibility for the other (Moreno Márquez 1987). In the postcolonial tradition, the positioning of a person or group as ‘central’ and ‘marginal’ prerequisites the political and discursive exclusion:

To successfully position a character as the Other demands the a priori binary construct of center and periphery, as discussion of the Other is impossible without a primary definition of the self, which, in turn, rests upon where we see ourselves located. If we inhabit the center of our existence (our world, life, knowledge), then the Other, who cannot inhabit the same place, becomes marginalized by definition: they cannot be us. They are different and apart from us. They are outside. (Kerslake 2010, 9)

Representing, narrating and negotiating both understandings of otherness is the genuine merit of particularly British and Canadian science fiction (Roberts 2006, 17; Seed 2011, 27), which transforms the concrete moral and political issues of time and place into an abstract ‘other.’ Within this process of transformation, spatial demarcations – like the topography in Midwich – represent previously constructed differences.

The children’s otherness is even marked textually: ‘the Children (now beginning to acquire an implied capital C, to distinguish them from other children’) (MC 103). This textual marker very much resembles the standpoint in Postcolonial Theory and Cultural Studies, where the pronounced ‘Other’ (with a capital ‘O’) refers to politically and socially unaccepted, marginalized, or excluded persons in order to “connote an abstract and generalized but more symbolic representation of empire’s ‘others’” (Ashcroft, Griffiths and Tiffin 2007, 156). This marker was later reflected precisely in postcolonial theory (Spivak 1985, 132). Within the novel, the textual marker of the ‘*C*hildren’ as opposed to ‘*c*hildren’ hints at the text’s dimension of social critique, characteristic of processes of ‘Othering,’ before – and that is actually the inspiring point and great merit of the novel – it was discussed academically.

In *The Midwich Cuckoos*, the children’s striking feature is their “abstract foreignness, not calling to mind any particular race, or region” (MC 148). By expressing their strangeness as “abstract” and “untraceable,” Wyndham narrates otherness in both the psychoanalytical and the political forms. The women’s “incubation” (65) by a foreign species touches upon the ground-breaking features of otherness the psychoanalytical notion: the children become an ‘other’ (a subject) through birth; and the peculiar conditions of pregnancy, birth and infancy, which shape the villagers’ attitudes, result in a status of the Other, which is morally and politically different from the inhabitants.

The children’s maturity process is characterized by a collective experience of their Otherness as it is constructed by the Midwichers. They encounter a social non-acceptance by being called “intruders” (106), “invaders” (188) and even “monsters” (167). Here, it is important to keep in mind that Wyndham’s works can be characterized as ‘logical fantasies.’ By mentioning the Children’s “abstract foreignness” (148), *The Midwich Cuckoos* is a logical fantasy on Otherness in its multiple forms.

Even though the Midwich scenario might appear very far-fetched at first, the children’s ‘monstrosity’ seems almost commonplace when viewed against the historical background of the pregnant unmarried women in the 1950s (Hanson 2004, 144). In one interpretation, *The Midwich Cuckoos* negotiates the social status of so-called ‘bastards’ in post-war rural Britain, which were – like their mothers’ – highly stigmatized and socially excluded. It is, for this reason, no less than Margaret Atwood (2015), who writes:

In my opinion, Wyndham’s chef d’oeuvre is The Midwich Cuckoos; it was published in 1957, just as I was 17 and going off to university, so it was a good time for me to be thinking about the consequences of being impregnated by an alien while unconscious, then giving birth to an alien species that ruins your life. The Midwich Cuckoos was certainly a graphic metaphor for the fear of unwanted pregnancies as experienced by the teenage girls of that pre–birth-control era. I myself had a dream about a highly intelligent nonhuman baby after reading this book, although the infant was green; so Wyndham must have been connecting strongly with the collective unconscious.

This contextual interpretation illustrates that Wyndham’s novel can be seen as a moral standpoint concerning the social status of the ‘Other’ in that it problematizes its social status. Likewise, Midwich turns into a microcosm cut off from its surroundings. Other villages in this area see it as “a kind of mental home without bars” and a “local deficiency area” (133) to other villages. Compared to the neighboring villages, Midwich is considered the Other; the uncanny, strange and monstrous counterpart. However, through Richard’s point of view, it also becomes evident that Midwich’s perception as “daytouched” (133), i.e. affected by the ‘Dayout,’ corresponds to a perspective from the outside – the narrator, being part of the village, hardly finds anything strange in the people’s behavior. This, in turn, puts the reader in an ambivalent state because objectively the village is unusual, but the reader is drawn in due to the subjective, narrative perspective.

After the children’s birth, the negotiation of Otherness continues when they perform telepathy and make people do things they otherwise would not do. For example, all children who were born outside Midwich are brought back to the place even if their mothers do not want to move. Even though the village mistrusts the children, it comes to accept their existence in the end. Although they finally decide to give birth to the children, doubts remain whether their decision is a right one as it is commented by Gordon Zellaby, the village’s novelist and the most educated inhabitant: “How is one to know with – strangers?” (167).

The ambivalence prevails when the children are later offered to live at The Grange as “a group of their own kind” (132). Since their perspective is never a part of Richard’s narration, the reader remains clueless with regard to the concrete events in The Grange. Richard only imparts that at The Grange, Zellaby educates the children. The children’s collective position at this special place nearly turns out to be their fate in the end when Midwich decides to burn down The Grange. By using their telepathic capacities and ordering Midwich’s inhabitants to attack one another, the Children rescue themselves. Moreover, they isolate the community from the outside and stop all residents from leaving the village. This leads to incomprehension on the part of non-Midwichers; a bus driver, who observes passengers being inhibited from boarding by an invisible object, says: “In Oppley they’re smart, and in Stouch they’re smarmy, but Midwich folk are just plain barmy” (168).

Likewise, the characters show attitudinal ambivalence towards the Children alternating between curiosity and suspicion or even fear while the reader is uncertain as to the reasons behind these events as Richard offers no explanation. Nevertheless, the vagueness of their actions is maintained since the children prevent neither the police from “coming inside” nor the ambulance from bringing the injured to hospitals, i.e. “the outside” (173). So, despite their peculiarities, the Children seem to share some sort of moral compass or intuition.

## ‘C’ as in ‘clone’

The events in Midwich also attract the military, as the Intelligence Major assumes that the place is attacked by the opponent of its time, i.e. the “Ivans” (38), or an alien force. Accordingly, in contrast to the press and some villagers, the military is not interested in The Grange but rather focused on “a large dent in the ground which certainly looked as if something massive had rested there for a while” (47). Although it is not spelled out within the novel, this incident hints at the landing of a U.F.O.

Like the narrator, Richard, the novelist, Zellaby, has a special position in Midwich society since he is the one who tries to explain the pregnancies and gets closest to the children. In various references to novels and theories, Zellaby is portrayed as a very well- educated character, while other villagers and, especially, Bernard Wescott dismiss his explanations. Along with the village’s doctor, Zellaby tries to find a rational explanation for the pregnancies, which differ from the U.F.O. hypothesis:

[I]t is possible, is it not, in some of the lower forms at any rate, to induce parthenogenesis?’ ‘But not, as far as is known, among any of the higher forms – certainly not among mammals.’ ‘Quite. Well then, there is artificial insemination.’ ‘There is,’ admitted the doctor. ‘But you don’t think so.’ ‘I don’t.’ ‘Nor do I. And that,’ Zellaby went on, a little grimly, ‘leaves the possibility of implantation, which could result in what someone – Huxley, I fancy – has called ‘xenogenesis’. That is the production of a form that could be unlike that of the parent – or, should one perhaps say, ‘host’? It would not be the true parent. (63f)

The characters refer to those reproductive medical procedures, which correspond to the state of research in the mid-twentieth century: First, the phenomenon of parthenogenesis, and second artificial insemination, which was already practiced in the 1950s (Schreiber 2007, 153–187). The first option is considered unlikely since parthenogenesis is not known to happen in higher mammals, such as the *homo sapiens*. The possibility of artificial insemination is also dismissed because all women in Midwich must have ovulated at the same time: “By the law of averages it simply is not possible in any sizeable group of women taken at random, for more than twenty-five per cent of them to be in the same stage of pregnancy at the same time” (MC 64). Methods, such as a “fertilizing gas” (83), are also discarded by Zellaby. According to him, one potential but very frightening explanation is a phenomenon that Thomas Henry Huxley (1908), the grandfather of the novelist Aldous and biologist Julian Huxley, called *xenogenesis*, referring to the growth and birth of an ‘alien’ species in the body of another. This possibility makes the village extremely anxious. The employees at The Grange, in turn, maintain their silence: “Either the researchers were of the opinion that the affair somehow came within the compass of their oaths of secrecy, or else they were of the opinion that it was safer to act as if they did” (MC 77). The text leaves open (until its end) who initiated the xenogenic pregnancies. Possible answers in the diegetic world are the researchers – staging the U.F.O. scenario to anesthetize and distract the village – or an actual U.F.O. landing, which would surely categorize *The Midwich Cuckoos* as a fantasy novel (for this interpretation, see Hanson 2004, 144).

In fact, it is precisely the text’s vagueness, which classifies *The Midwich Cuckoos* as logical fantasy in the style of Wyndham (see above). Despite some indications in the text, the alien invasion being the cause of the inexplicable pregnancies remains just one interpretation among others. Considering the overall symbolism of the novel, there is another explanation for the events, namely that *The Midwich Cuckoos* is one of the first novels on human cloning. Thus, I propose an interpretation that these fictional pregnancies resulting in children, who all look and behave the same, evoke techniques of reproductive medicine, which was growing as an academic discipline after WWII (Franklin 1997, 101–130; Hanson 2004, 128–137). From the narrated actions and events, this interpretation is not evident, but some parts of the novel and some contextual information support the hypothesis that *The Midwich Cuckoos* is one of the first novels on human cloning (see also Nerlich et al. 2001).

For some readers, this might come as a surprise since the technical possibility of cloning – transferring the nucleus DNA of any somatic cell from a full-grown mammal into an egg cell previously deprived of its nucleus DNA – is often connected to the birth of the sheep, Dolly, decades later (Wilmut et al. 1997). However, the history of cloning can be traced back much longer, as it was already part of basic research in the post-war period. As the application to mammals was very far-fetched, the goal was to obtain differentiated knowledge of cell development. Briggs and King (1952) at the Cancer Research Institute in Philadelphia wanted to specify the cells at a certain stage of development of the embryo. By Spemann’s (1938) results, it was already known that eight-cell embryos are totipotent. However, it was still unclear what development would lead to a later differentiation led by cells:

As Spemann was aware, cellular fates might be sealed in at least two ways. The identity of individual cells might be established by their local environment – their positions relative to other tissues. But it was also possible that during development the nucleus underwent changes that progressively stifled its developmental capacity, making it less than totally potent. (Gurdon 2001, 41)

First of all, researchers were eager to find out whether there were different cell nuclei in the embryo or whether the differentiation during the development process happened after fertilization. (Today, we know that the latter one is true.) For their experiment, Briggs and King gutted eggs from a frog larva and put them in the nuclei of a blastula, a very early embryonic stage of development. New larvae resulted from this nuclear transfer. Furthermore, nuclei from the gastrulation stage, the next step in embryonic development, were also transferred. From these cells, however, viable larvae were developed (Heinemann 2005, 169). By this, Briggs and King proved that different cells of the embryo had different genetic material. However, there was still an uncertainty because, due to technical circumstances in these experiments, not only the nucleus but also a small part of the cytoplasm was transferred. Following Briggs and King’s research, never living tadpoles emerged: “The implications for any proposal to clone from an adult cell are clear: Dolly was, by the understanding of the 1950s, a fiction, a mirage, an impossibility” (Wilmut/Highfield 2006, 57).

Although the transfer itself was successful, most attempts did not lead to cell division (Gurdon 2001). Assuming that this was due to technology, the embryologists henceforth used cells from the gastrula, which were developed even further. However, a contradiction arose: Many embryos did not develop or had severe anomalies. However, about twenty percent of the attempts were successful although with the most differentiated cells. Briggs and King had previously declared this to be impossible (1952).

After American research left many questions unanswered, it was John Gurdon (born in 1933) in England, who continued the basic research on cloning. In fact, it was in Great Britain, where Dolly was born in 1996. Already in 1958, Gurdon succeeded in Oxford by creating not only tadpoles through the transfer of embryonic cells but also frogs. It was this research, which later refuted the theses of Briggs and King (Gurdon 2001, 45).

One link between *The Midwich Cuckoos* and the cloning experiments of its time is plausible by considering the children’s features more closely. After being born, all of them are healthy and inconspicuous apart from their very fair hair and golden eyes (MC 148). The children’s golden iris is reminiscent of the eye of a frog that Briggs and King tried to clone at Philadelphia University, the *Rana pipiens*. Moreover, the children’s remarkable features do not only include their eyes and telepathic capacities but also their resemblance to one another: “Sixty-one similars – so similar that most of their ostensible mothers cannot tell them apart” (MC 100). The perception of the children as ‘another species’ is not only suggested by their resemblance but also by their behavior as they learn, act, and talk in a similar manner. Hence, they share a collective identity, conscience and phenotype, even though they are all different individuals:

[I]f I ask him to perform an action, I shall get more or less the same result, but it is likely to be more successful with some who happen to have better physical coordination than others – though, in point of fact, with such close similarity as there is among the Children the variation will be small. [...] [I]t will not be an individual who answers me, or performs what I ask, it will be an item of the group. (122)

Even though the technique of cloning is not mentioned in the novel, the topoi of sameness and collective identity of a special group that dissociates from others, historically align with a list of cloning novels (Nerlich et al. 2001).^5^ So, they evoke clones in a specific way.

The Children form a special group of their own; talking and playing only with each other and rarely with other children in Midwich. Even Zellaby utters doubts about the U.F.O. thesis, assuming that the events and experiments at The Grange led to the pregnancies:

One has to speculate, of course. Not very satisfactory, I’m afraid, and sometimes uncomfortably. It is, for instance, disquieting for a good rationalist, such as myself, to find himself wondering whether perhaps there is not some Outside Power arranging things here. When I look round the world, it does sometimes seem to hold a suggestion of a rather disorderly testing-ground. The sort of place where someone might let loose a new strain now and then, and see how it will make out in our rough and tumble. Fascinating for an inventor to watch his creations acquitting themselves, don’t you think? To discover whether this time he has produced a successful tearer-to-pieces, or just another torn-to-pieces and, too, to observe the progress of the earlier models, and see which of them have proved really competent at making life a form of hell for others. [...] Ah, well, […] the speculations tend to be uncomfortable. (MC, 204/205)

Through its most educated character, Zellaby, the text again represents an uncertainty of the events in Midwich as well as the possibility of human intervention, an experiment. As a ‘testing-ground,’ The Grange might play a role inducing the experiment out of scientific curiosity, as Zellaby continues: “And when I say ‘Inventor,’ I don’t necessarily mean an individual, of course. It seems to me that if a team of our own biologists and geneticists were to take a remote island for their testing-ground they would find great interest and instruction in observing their specimens there in ecological conflict” (205).

Since Zellaby is presented as the most rational character, the interpretation of *The Midwich Cuckoos* as an early novel of reproductive cloning, or at least as an evocation of cloning, seems plausible. Even though it remains unclear in the conclusion of whether or not the children were the result of aliens or human experiments, the many allusions to medicine, biology, and genetics offer an interpretation yet unconsidered: It is reasonable to suggest that the insular village of Midwich refers to experiments that were first possible in the 1950s when technological progress formed the basis for cloning.

Besides, *The Midwich Cuckoos* is not the only one of Wyndham’s works, which draws upon cloning. *Plan for Chaos* (posthumously published in 2009)^6^ is another instance of Wyndham’s use of a version of cloning, which narrates how Nazis are cloning an Aryan race. According to Ketterer (2009), who edited the novel, it was the first, which “deals with the theme of cloned Nazis.” Here, clones are on a mission to dominate and control the world; a salient theme in Wyndham’s oeuvre. Indeed, *Plan for Chaos* can be seen as a companion piece to *The Midwich Cuckoos*.

## Negotiating the utilitarian standpoint

Like the war memorial and the green triangle, the shape of The Grange is highly symbolic. The descriptions of the building are significant on an interpretative level: “[U]tilitarian wings […] were added to The Grange when the Ministry took it over for Research” (MC 12). On the one hand, this shows that the building is complemented by additional space in the form of laboratories (215). The original agricultural function of the space is exceeded with the addition of ‘wings.’ On the other hand, the word ‘wings’ refers to the context of theater where ‘wings’ are the spatial scenery and background of the stage. In this sense, the fictional ‘utilitarian wings’ in the novel can be interpreted as the cultural background of the villagers’ attitudes, actions and aims. Herein lies a reference to the British cultural tradition of utilitarian ethics. In this sense, I interpret the ‘utilitarian wings’ as a pragmatically larger building and, in parallel to the novel’s symbolic dimension, as connected to ethical traditions that follow moral principles, such as the collective happiness of all people concerned by an action (Mill [1871] 2010, 50).

In Britain, Jeremy Bentham (1748–1832) and John Stuart Mill (1806–1873) founded utilitarian ethics. They still form an important and influential tradition of moral theory and bioethical evaluations of technologies, as in the work of the Warnock-Commission that scrutinized the rise of new reproductive technologies in Britain (Warnock 1987, 1998). Utilitarianism is a consequentialist form of ethics, which aims at the prevention of suffering and the increase of happiness. Classic utilitarianism refers to the consequences resulting from actions to morally evaluate them; that is, it promotes actions providing happiness and condemns actions providing suffering (Singer 2011, 3).

Although the consequences of actions are also relevant to other ethical theories, classical utilitarianism especially follows the common good as its main principle. The discussion around utilitarianism is, in fact, only a continuation of a cultural discourse that had long been present in British literature. For instance, More’s *Utopia* ([1516] 2010) already dealt with the question of how to achieve the greatest common good. Whereas *Utopia* was written in the Renaissance and was located insularly, the modern utilitarian discourse is oriented toward the future asking how the common good can be achieved (Macho 2005).

Reading *The Midwich Cuckoos* from an ethical perspective, we see how moral premises underlying utilitarianism emerge. Since the military knows that a similar incident occurred in Russia, it observes Midwich to ascertain whether this form of life consists of artificial intelligence (MC 190). When the Russian village is destroyed, the British military sees the children as a threat to the human species. The treatment of children in Russia finally leads to a contrast of two different systems: Russia, where the individual is subordinated to the state, and England, which considers the state an institution that serves individuals (198). Since the text mentions the “iron curtain” (189), the fear of strangers seems to be a negotiation of the cold war and the invasion of the Soviets. Thus, *The Midwich Cuckoos* also negotiates collective processes of Othering between the East and the West after WWII.

Implicitly, Zellaby contrasts England’s civilization with negative eugenics of National Socialism as he comments: “History has shown us to be more tolerant of minorities than most” (198). This statement, in turn, is contrasted with a claim by the children, who argue that even a civilized country like England could probably not tolerate a minority that one day might become too powerful and take over control (198). Through the children’s reflection, the situation becomes a political and moral issue: “They [left-wing forces] want to defend our rights as a threatened minority, and children, and that. Their leaders will glow with righteousness on our behalf. They will claim, without referendum, to be representing justice, compassion, and the great heart of the people” (199). Thus, the children are seen as a “national danger” (191) and become an ‘Other’ by being considered a “racial danger” (191).

This political negotiation, as developed by the children themselves, is also mirrored in the village. Some people in Midwich argue that the children are “another species” (158), leading then to conclude that killing the children does not count as murder since “murder is, by definition, the killing of one’s *own* kind” (158). Thus, their elimination is based on the necessary premise of Otherness:

At present, we are conceding them all the privileges of the true *homo sapiens*. Are we right to do this? [...] [A]re we not fully entitled – indeed, have we not perhaps the duty? – to fight them in order to protect our own species? After all, if we were to discover dangerous wild animals in our midst our duty would be clear. (158)

In this scene, Wyndham develops the central moral standpoint of the novel by expressing a harsh critique against instrumentalizing the argument of Otherness by legitimizing their elimination. Considering a strategy of self-defense, the majority favors killing them by regarding the children as savages without morals and rationality. On the contrary, the minority still considers a dialogue with the children in order to find a solution through deliberation. The children are – even if the term is not mentioned in the novel – *outlaws* lacking any juridical title. As a person of character, it is Zellaby who acts as their lawyer and moral advocate: “[I]t would appear that if they kill us it is murder, but if we were to kill them it would be just something else. One cannot help feeling that a jurist, lay or ecclesiastical, would find such a proposition ethically unsatisfactory” (158). In this scene, Zellaby understands morals as a protective function for the social ‘Other:’ If the children actually are ‘Other species’ and if murder only entails the killing of ‘one’s own kind,’ they cannot ‘murder’ the people in Midwich either.

In the end, the children ironically point out “a moral dilemma of some niceness” (208): They cannot be killed without harming the village since they would always anticipate and counteract any such an attempt due to their telepathic capacities, whereas keeping them alive might bring harm to the village. Therefore, their existence not only raises the question of how to act but also whether the killing of some villagers is necessary (or even morally legitimate) to save the rest of Midwich and eliminate the children. Again, Zellaby provides a solution: “In a quandary where every course is immoral, there still remains the ability to act for the greatest good of the greatest number” (158). This allusion to utilitarianism places the novel itself in the British tradition and makes it a literary form of classical thought experiments. These, in turn, as fictional as *The Midwich Cuckoos*, are often used to illustrate varying moral principles as well as the difference between intentional harm and the side effects of a harmful action.^7^

In the novel, Zellaby, who is terminally ill, not only reflects the dilemma but also acts according to his analysis. By committing suicide, he kills the children, an application of the utilitarianism: ‘the greatest good for the greatest number,’ i.e. the survival of the majority of Midwich’s population and the elimination of the threat. However, he acts on mere assumptions since it remains unclear whether the children would have been a real danger to society. As Richard, the narrator, helps Zellaby transport the film equipment and candy, he approaches the children as *children*, for the first time:

For the first time since my return I was able to appreciate that the Children had ‘a small c’, too. [...] It was impossible to associate the Children, as I saw them now, with danger. I had a confused feeling that these could not be the Children, at all; that the theories, fears, and threats we had discussed must have to do with some other group of Children. [...] I was finding it harder every moment to believe that we had not all of us been somehow deluded by a sweeping misunderstanding about the Children[.] (MC 216)

While mostly validating utilitarian ethics, Wyndham still manages to depict the children not as monstrous but as harmless, curious and friendly. During a daily encounter, Richard eventually transcends his own attitude, which makes the seemingly utilitarian act appear even more like a mere genocide. Similarly, the ambivalence between scientific experiments and an extraordinary event, such as the landing of an U.F.O., is never completely resolved in *The Midwich Cuckoos*. Instead, the narrator, who observes the village intensively, constantly perpetuates it.

## Conclusion

With the final change in Richard’s point of view, the story ends on an ambiguous note, which makes room for an important ethical reflection; that is to say, to what extent is our moral judgment influenced by our experiences of prior assessments of ‘Others’? Does our appreciation and evaluation of the ‘Other’ have any influence on our moral judgement (Hansen and Cronjäger 2015)? In this paper, I proposed a strategy of novel reading that first and foremost opens up a perspective on science fiction for the discourse on science and technology with a critical perspective on otherness and othering. It is crucial to consider novels such as *The Midwich Cuckoos* not as a mere fiction but to take them seriously given their moral standpoint.

Typically, Wyndham’s ‘logical fantasies’ closely follow the events of the characters who, given the background, fall victim to a technological or scientific constellation. In doing so, he disregards the ethical problems of science and technology and, instead, discusses related social consequences. Moreover, his reproductive fantasy offers leeway for a wider reflection of the historical and social construction of otherness in the context of reproduction, e.g. the status of so-called ‘bastards’ or social taboos with regard to reproductive techniques.

Although each clone has a name – significantly marking them as individuals – the village mainly refers to them as ‘baby’ or ‘child’ since they are indistinguishable, at least for outsiders. However, from a perspective of otherness, the following reading strategy is plausible: They Children are perceived as ‘Other’ (a threat) in the political notion, and not as ‘other’ (a person) in the psychoanalytical sense. By seeing them as one threatening, strange, and uncanny entity, Midwich encounters the Children as one person, one individual. However, through the narration, it becomes clear that the development of their collective identity is a consequence of a very similar, if not the same socialization process, during which the villagers discursively construct their Otherness, which is then followed by fear. With these alternations and symbolism of Othering, *The Midwich Cuckoos* is not a dystopia, such as Huxley’s *Brave New World.* Rather, it may count as a literary thought experiment focusing on a collective microcosm and its negotiation of unexpected events and persons. For medical humanities, novels such as *The Midwich Cuckoos* might provide an ethical perspective yet unconsidered. Particularly with the spatialized dynamics of inclusion and exclusion, *The Midwich Cuckoos* confronts its readers with a perspective of society’s ‘Others’ and the relevance of a community’s attitude when evaluating their status.

## Endnotes

^1^ His full name was John Wyndham Parkes Lucas Beynon Harris. When writing fiction, he chose to be referred to only as John Wyndham after WWII. For an analysis on John Beynon Harris’ pseudonym within the context of differences between British and US-American science fiction, see Sweet (2018).

^2^ The story about carnivorous, mobile, and very aggressive plants, which arrived on Earth as spores from a meteor shower, was made into the 1962 feature film of the same name, three radio drama series (in 1957, 1968 and 2001) and two TV series (in 1981 and 2009).

^3^ In the following, I will abbreviate the novel as MC and quote directly from it in the text.

^4^ On the history of abortion in before the enactment of the Abortion Act in 1967, see Brooks (1988).

^5^ The most famous prequel is Aldous Huxley’s ([1932] 1985) *Brave New World* and the most famous sequel probably is Naomi Mitchisons *Solution Three* ([1975] 1995). For a full analysis of *The Midwich Cuckoos* in the history of cloning, see Hansen (2016).

^6^ According to Ketterer (2009), it was written during the same time as *The Day of the Triffids *(which was published in 1951) and completed shortly after.

^7^ For the discussion of classical examples, such as the first version of the so-called trolley experiment, cf. Foot (1967).
